# Vesico-Vaginal Fistula Successfully Treated by the Suture

**Published:** 1831

**Authors:** 


### XVII.

### VESICO-VAGINAL FISTULA SUCCESSFULLY TREATED BY THE SUTURE.

MARIE REGGIANI, æt. 22, had laboured under a vesico-vaginal fistula since her first confinement, which had been protracted and severe; the finger could be readily passed through the fistula into the bladder. For eight months a great variety of plans were employed without success, and she repaired to M. Malagodi at Bologna, who performed the operation we are about to describe. Having placed the patient in the lithotomy-position, he introduced the fore-finger of the right hand, covered with the finger of a glove, into the fistulous opening, and bending it drew down the left border of the fistula to the orifice of the vagina; and then, with the other hand he pared off the border with a straight bistoury. The hand was changed, and the same operation repeated on the right border of the fistula. The object was now to bring the two pared edges in contact. M. Malagodi had armed each end of these ligatures with a very small curved needle, and there was a handle to which the needles might be fixed at pleasure. He then re-introduced the right index finger into the opening, and brought down its left border, and with the left hand passed a needle through it near the posterior angle of the wound. A second and third needle were passed in the same manner at equal distances from each other, and their opposite border was transfixed in the same manner, when the ligatures were tied, and the sides of the opening brought together. The patient was placed on her back in bed, a catheter kept in the bladder, and the urine allowed to flow constantly into a porringer. Until the morning of the third day the water was passed through the catheter only, but then it began to escape by the wound into the vagina. On the fourth day an examination was made by M. Malagodi, who found that the two posterior sutures remained firm, and on withdrawing them the union of the sides of the fistula was complete. The anterior ligature, on the other hand, had torn the left border of the wound, and nearly a third of the original fistula thus remained unhealed. To effect the contraction and perfect cicatrization of this, M. Malagodi touched the edges regularly with the nitrate of silver, leaving the catheter constantly in the bladder. The plan was persisted in for some weeks, and was crowned with perfect success.—*Raccoglitor Medico, July* 1829.

---

